# Structural Analysis of Oxidized Sucrose and Its Application as a Crease-Resistant Crosslinking Agent

**DOI:** 10.3390/polym14142842

**Published:** 2022-07-13

**Authors:** Mao Feng, Xiaodong Hu, Yingting Yin, Yajing Liang, Jiarong Niu, Jinbo Yao

**Affiliations:** School of Textile Science & Engineering, Tiangong University, Tianjin 300387, China; xgf@msn.com (M.F.); easterhu@163.com (X.H.); 15527367472@163.com (Y.Y.); lyjbang@foxmail.com (Y.L.)

**Keywords:** oxidized sucrose, structural analysis, hemiacetal, hydrate, cotton fabric, crease resistant

## Abstract

Oxidized sucrose is a non-formaldehyde crosslinking agent with many applications in polymer crosslinking and modification, such as in the preparation of starch films and protein films. However, research on the structure of oxidized sucrose is lacking. In this paper, oxidized sucrose was synthesized through selective oxidation of sodium periodate. By LC-MS, FTIR, TGA, NMR, and HRMS analyses, it was shown that oxidized sucrose existed in the form of a hydrate, and the tetraaldehyde oxidized sucrose could isomerize into the form of two six-membered hemiacetal rings. The structure of oxidized sucrose was also verified by theoretical calculations. Furthermore, the diffusional properties of oxidized sucrose were investigated by the rolling-film method. Finally, it was found that oxidized sucrose used as a crosslinking agent could effectively improve the wrinkle recovery performance of cotton fabrics.

## 1. Introduction

Cotton fabrics wrinkle easily after wearing and washing, causing many inconveniences. In order to maintain its flat appearance, a crease-resistant finishing of cotton fabrics has been widely used. At present, most finishing agents with hydroxymethyl groups, such as DMDHEU, release formaldehyde during finishing and wearing [1,2,3]. Therefore, researchers have developed many non-formaldehyde finishing agents, such as dialdehydes, polycarboxylic acid, and polyurethane. Unfortunately, most new crosslinking agents are not as effective as DMDHEU. Among them, research on dialdehydes mainly focuses on glyoxal and glutaraldehyde [4,5,6,7,8,9,10,11]. Although the crease resistance of cotton fabrics finished with glyoxal and glutaraldehyde is satisfactory, dialdehydes with low boiling points are strongly irritative during finishing. Due to their high reactivity and volatility, they are prone to react with proteins. Therefore, small-molecule aldehydes are usually toxic and carcinogenic [12,13,14].

Sucrose is widely distributed in plants and is non-toxic and renewable. Saccharides were oxidized to polar or polymeric aldehydes, which exhibit low toxicity and potentially high reaction efficiency. There are many studies on oxidized sucrose used as a biological cross-linking agent, for example, cross-linking of starch and protein to obtain starch films or protein films [15,16,17,18,19]. Since it contains multiple hydroxyl groups, oxidized sucrose can also improve the hydrophilic properties of cross-linked polymers. However, previous studies [15,16,17,19,20,21,22,23,24,25,26] focused on the application of oxidized sucrose and seldom on its structure. The few examples of characterization of oxidized sucrose, were mainly based on FTIR and NMR, which only showed that there were aldehyde groups in the product and never proved whether the oxidized product was a typical oxidized sucrose molecule with four aldehyde groups. After analyzing cured oxidized sucrose at 160 °C by mass spectrometry, Lou (2019, 2021) proposed that the self-polymerization of oxidized sucrose decreased its cross-linking effect of cellulose macromolecules. In fact, intermediate products of sucrose oxidized by periodate may form, which may be converted to other structures in general circumstances, such as hemiacetals and hydrates [27,28].

Compared with most cross-linking agents, the cross-linking reaction of oxidized sucrose with polysaccharides has lower activation energy. For example, the activation energy of the reaction of oxidized sucrose with starch is only 33.22 kJ/mol [17], while those with glyoxal and glutaraldehyde are 270 kJ/mol and 262 kJ/mol, respectively, and that with citric acid is 48.65 kJ/mol [29]. The activation energy of the cross-linking reaction of BTCA with cellulose is 52.9 kJ/mol [29]. Polycarboxylic acid finishing agents, such as BTCA, are good cross-linking agents. However, they require a phosphorus-containing catalyst, which causes environmental pollution.

Oxidized sucrose should have good cross-linking properties based on the lower activation energy of the cross-linking reaction. Theoretically, there are four aldehyde groups in oxidized sucrose, and the number of aldehyde groups that can participate in the cross-linking reaction is higher than that found in other cross-linking agents. However, as an anti-wrinkle reagent for cotton fabrics, oxidized sucrose could not achieve the expected cross-linking effect. Therefore, we carried out a detailed study of the chemical structure of oxidized sucrose, hoping to find the reason for its poor cross-linking effect when applied to cotton fabrics. Furthermore, unlike starch and proteins, oxidized sucrose solutions and cotton fabric are heterogeneous systems. Therefore, the diffusion of oxidized sucrose into cotton fibers should be analyzed. In this study, besides the FTIR and NMR characterization of oxidized sucrose, we also performed LCMS, HRMS, and TGA analyses. Theoretical calculations were also used to provide a theoretical basis for the characterization results and applications of oxidized sucrose. Finally, oxidized sucrose was used as a crosslinking agent for the crease-resistant finishing of cotton fabrics.

## 2. Materials and Methods

### 2.1. Materials

Sodium periodate, barium chloride, sodium hydroxide, magnesium chloride, aluminum chloride, strong acid polystyrene cation-exchange resin, and strong base polystyrene anion-exchange resin were purchased from Tianjin Kemiou Chemical Reagent Co., Ltd. (Tianjin, China). The Schiff’s reagent was purchased from Beijing Solarbio Science & Technology Co., Ltd. (Beijing, China). Cellulose membranes were purchased from Viskase (Lombard, IL, USA). All chemicals were of analytical grade and commercially available. Bleached cotton fabric (warp rib weave, 161 warp yarns and 76 wefts per 2.54 cm, weighs 140 g/m^2^) was purchased from Lutai Textile Co., Ltd. (Zibo, China).

### 2.2. Synthesis

**Figure 1:**
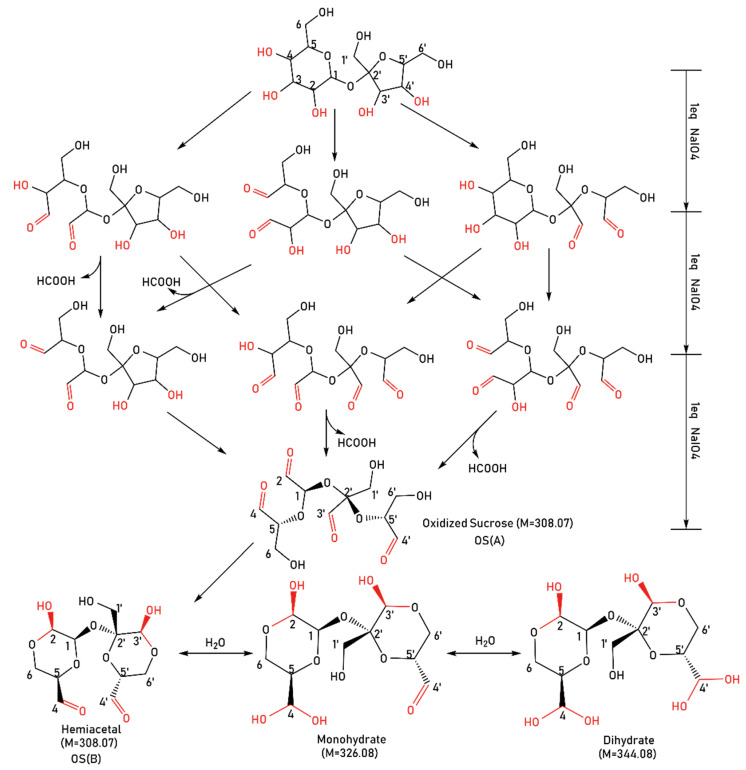
Reaction pathways for oxidized sucrose preparation.

Under constant stirring in the dark at 10 °C, sucrose (6.85 g) was dissolved in deionized water (100 mL), and sodium periodate (12.83 g) was added in 5 portions evenly and then stirred for 20 h. During this process, a sodium hydroxide (c = 5 mol/L) solution was added to maintain the pH at approximately 4.0. Then, barium chloride (6.4 g) was added to remove the sodium iodate in the solution. An aqueous solution sample was obtained, and the resulting oxidized sucrose was in the tetraaldehyde form, designated as OS(A). In order to remove ions from the solution, the obtained solution was repeatedly passed through a strong acid polystyrene cation-exchange resin column and a strong base polystyrene anion-exchange resin successively. Finally, an oxidized sucrose powder sample was obtained through freeze–drying and was dried at 60 °C for 10 h. The sample was stored in a desiccator for later use. The oxidized sucrose was in the hemiacetal form, designated as OS(B). The reaction pathway for the preparation of oxidized sucrose was shown in Figure 1.

### 2.3. LC–MS

LC was performed with an Accucore C18 column (100 mm × 2.1 mm, 2.6 μm) with a 0.22 µm pre-column filter on a Dionex Ultimate 3000 UHPLC system (Thermo Scientific, Waltham, MA, USA) at 30 °C. The mobile phase consisted of H_2_O with 0.1% formic acid (A) and acetonitrile (B). For oxidized sucrose OS(A) analysis, the gradient procedure based on mobile phase A was as follows: 0−0.5 min holding, 95%, 0.5−6 min from 95% to 5%, 6−7 min holding, 95%, 7−7.1 min from 5% to 95%, and 7.1−100 min holding, 95%. The flow rate was 0.3 mL/min.

The Q Exactive MS instrument (Thermo Scientific, Waltham, MA, USA) was operated in negative mode at a mass range of 55−825 *m/z*. Ionization of the analytes was achieved using negative electrospray ionization (HESI) to yield the target ions [M-H]^−^. Mass spectrometer parameters were as follows: sheath gas rate, 30 mL/min, capillary temperature, 320 °C, auxiliary gas temperature, 300 °C, spray voltage, 3200 V, and mass resolution, 35,000.

### 2.4. HRMS

Oxidized sucrose OS(B) was dissolved in HPLC-grade methanol. The molecular weight of oxidized sucrose was measured on a Bruker solanX 70 FT-MS (Bruker, Regensburg, Germany) with the ESI resource instrument in positive ion mode.

### 2.5. Thermogravimetric Analysis

Thermogravimetric analysis (TGA) was carried out on a Netzsch STA 449F5 (NETZSCH, Selb, Germany) in the temperature range of 30 °C to 1000 °C, at a heating rate of 10 °C/min in a nitrogen atmosphere using an Al_2_O_3_ crucible with 14.46 mg mass of the oxidized sucrose sample OS(B).

### 2.6. FTIR Spectroscopy

The oxidized sucrose sample was dried for 1 h at 140 °C. A FTIR spectrum was collected at a resolution of 4 cm^−1^ for 32 scans in the transmission mode (4000–400 cm^−1^) using a Nicolet iS10 (Thermo Scientific, Waltham, MA, USA) spectrometer at room temperature.

### 2.7. ^1^H-NMR

Oxidized sucrose samples OS(B) were separately dissolved in D_2_O and DMSO-d6, and sucrose was dissolved in D_2_O. In order to eliminate the interference of free water, the OS(B) samples were dried for 1 h at 140 °C. ^1^H-NMR and 13C-NMR spectra were recorded on a Bruker 400 MHz (Bruker, Regensburg, Germany) spectrometer.

### 2.8. Calculation and Theoretical Analysis

A conformational search was conducted using the GFN-xTB [30] program to obtain the stable conformations of oxidized sucrose. Geometry optimization and vibrational analysis were performed using ORCA 5.0.1 [31,32] at the B3LYP-D3(BJ)/def2-TZVP with the CPCM (water) theory, and single-point energies were computed at DLPNO-CCSD(T)/cc-pVTZ with CPCM (water). Finally, the lowest-energy conformer was chosen for analytical calculations.

### 2.9. Crease-Resistant Finishing of Cotton Fabric

Firstly, the cotton fabrics were immersed in the finishing solution, containing oxidized sucrose and catalysts, at 50 °C for 1 h. The cotton fabrics were padded on a horizontal padding mangle after dipping and rolling them two times until 80% solution absorption, then dried at 80 °C for 3 min, and cured under specified conditions. In this experiment, the best finishing process was as follows: the concentration of the aluminum chloride catalyst was 20 g/L, pH 3, the content of oxidized sucrose was 150 g/L, the curing temperature was 140 °C, and the curing time was 330 s.

To investigate the diffusional behavior of oxidized sucrose in cotton fibers, rolls of cellulose film were immersed in the solutions, and formaldehyde, glyoxal, and glutaraldehyde were added separately for comparison. The cellulose films with aldehydes fully colored the same excess of Schiff’s reagent in closed centrifuge tubes for half an hour. Adsorption and desorption of aldehydes on cellulose were sufficient. PerkinElmer Lambda 750 (PerkinElmer, Waltham, MA, USA) was used to measure the optical absorbance of the colored Schiff’s reagent. The maximum absorption wavelength of the red-violet solution produced by the reaction of the four aldehydes with the Schiff’s reagent was about 560 nm.

## 3. Results and Discussion

### 3.1. Oxidized Sucrose Sample

**Figure 2:**
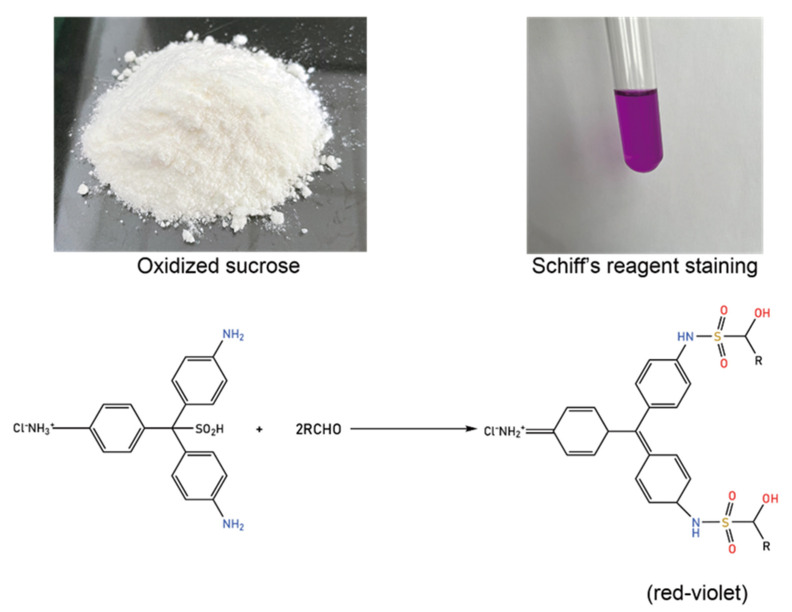
Reaction of the oxidized sucrose sample with Schiff’s reagent.

The oxidized sucrose sample OS(B) was a white powder, easily soluble in water, methanol, ethanol, and DMSO. As shown in Figure 2, the color of the aqueous solution of the sample turned red-violet after the addition of the Schiff’s reagent. It was proved that the sample contained aldehyde groups.

### 3.2. LC–MS and HRMS Analysis of Oxidized Sucrose

**Figure 3:**
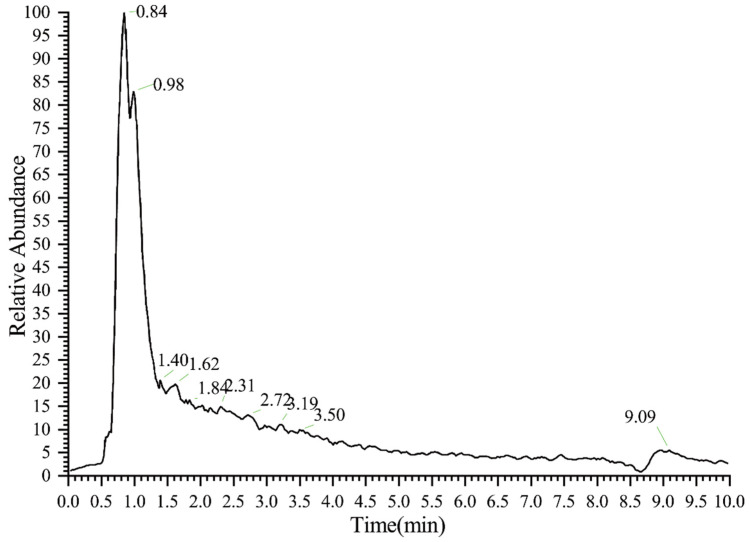
Base peak spectrum of OS(A) in negative ion mode.

**Figure 4:**
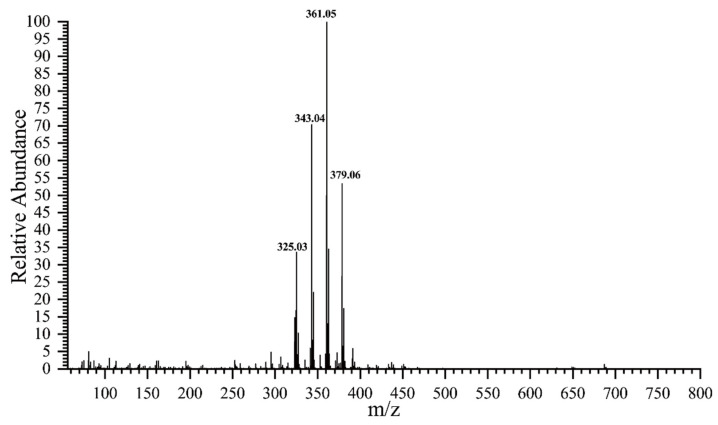
Mass spectrometry of OS(A), peak at 0.84 min in the base peak spectrum.

On the basis of the total ion current (TIC) diagram of the sample, the interference from the solvent and the mobile phase were relatively large, and the base peak (the strongest ion in the mass spectrum) spectrum (Figure 3) was selected. The peak at 0.84 min (Figure 4) with the highest relative abundance was chosen for analysis.

According to Figure 1, the ideal molecular mass (M) of the final oxidized sucrose (C_11_H_16_O_10_) is 308.07. Due to the negative ion mode, molecular-related ions were shown at *m/z* 325.03, 343.04, 361.05, and 379.06 in the mass spectrum Figure 4. The molecular weight difference between adjacent peaks was 18, equal to the molecular weight of the water molecule; therefore, thee peaks could be assigned to [M+H_2_O−H]^−^, [M+2H_2_O−H]^−^, [M+3H_2_O−H]^−^, and [M+4H_2_O−H]^−^, respectively. This MS result indicated the formation of hydrates of OS(A). This could be due to the different reactivity of the aldehyde groups, which led to the combination of a different number of water molecules with oxidized sucrose. Additionally, there were multiple hydroxyl groups and multiple aldehyde groups in each oxidized sucrose molecule, and the solution was weakly acidic. Oxidized sucrose could undergo an intramolecular reaction to form a cyclic hemiacetal. The presence of the hemiacetal could be one of the factors affecting the number of water molecules bound with oxidized sucrose.

**Figure 5:**
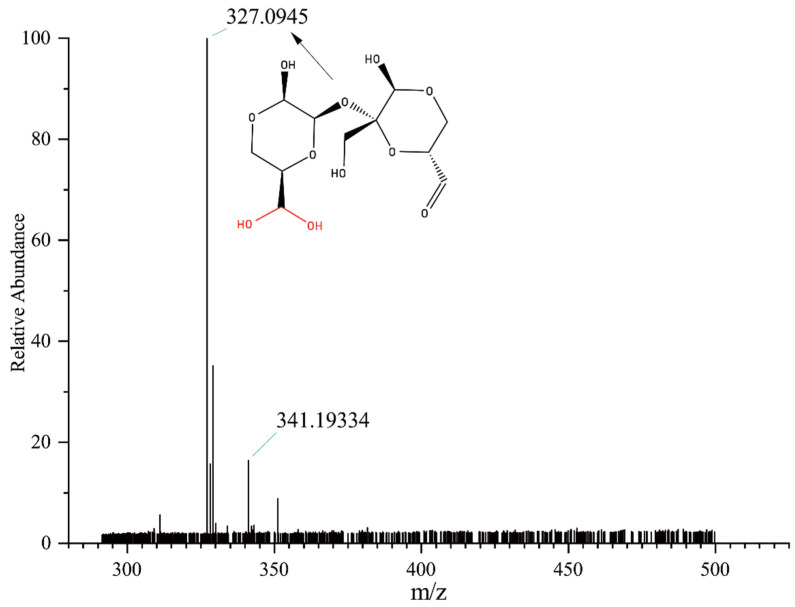
High-resolution mass spectrometry of OS(B).

Mass spectrometric analysis of OS(B) by HRMS is shown in Figure 5. In positive ion mode, the oxidized sucrose molecular-related ions [M+H_2_O+H]^+^ and [M+CH_3_OH+H]^+^ appeared at *m/z* 327.09450 and 341.19334. The HRMS results also showed that oxidized sucrose formed a hydrate (*m/z* = 327.09450).

### 3.3. Thermogravimetric Analysis of Oxidized Sucrose

**Figure 6:**
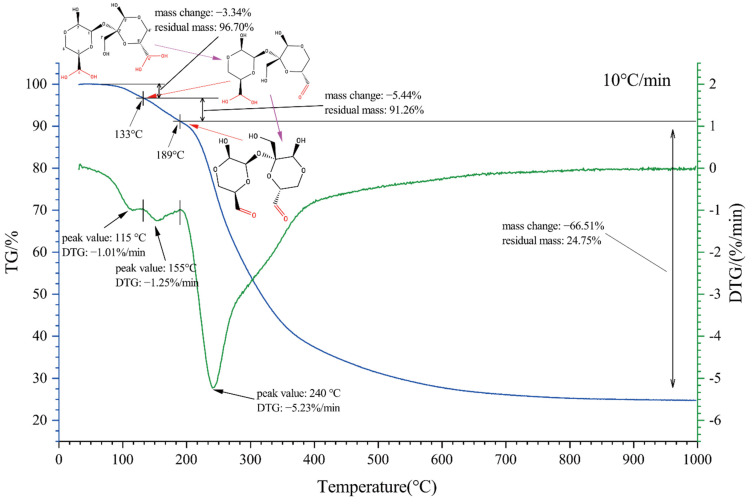
Thermogravimetric analysis of OS(B).

The results of thermogravimetric analysis (TGA) and derivative thermogravimetry (DTG) of OS(B) are shown in Figure 6. The sample pyrolysis process was divided into three stages. The 90–133 °C stage was mainly due to the loss of water molecules combined at C4′, with a residual mass of 96.70%. The residual product at this stage was the monohydrate of oxidized sucrose (C_11_H_16_O_10_·H_2_O). The second stage was 133–189 °C. In this stage, the weight of the sample was reduced by −5.44%, and the residual mass was 91.26%. The lost molecular weight was about 17.74, close to the loss of one water molecule from each oxidized sucrose molecule. Therefore, it was speculated that the mass loss in the second stage was mainly due to the loss of water combined at C4, and the residual substance in this stage was anhydrous oxidized sucrose (C_11_H_16_O_10_). Finally, oxidized sucrose kept pyrolyzing.

### 3.4. FTIR Analysis of Oxidized Sucrose

**Figure 7:**
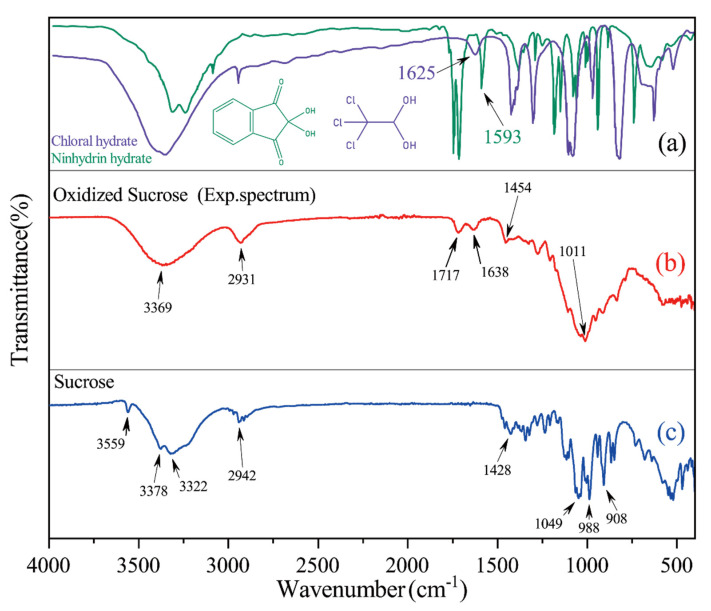
FTIR spectra of sucrose and OS(B) (**b**), sucrose (**c**), chloral hydrate and ninhydrin hydrate (**a**). Note: The chloral hydrate and ninhydrin hydrate spectra were obtained from the Spectral Database for Organic Compounds SDBS.

Figure 7 shows the FTIR spectra of sucrose and oxidized sucrose. The FTIR spectrum of sucrose (Figure 7b) showed O-H stretching vibrations at 3559 cm^−1^, 3378 cm^−1^, and 3322 cm^−1^, the C-H stretching vibration at 2942 cm^−1^, C-H bending vibrations at 1428 cm^−1^, and C-O-C stretching vibrations at 1049 cm^−1^, 988 cm^−1^, and 908 cm^−1^.

Compared with sucrose, the peak of oxidized sucrose at 3559 cm^−1^ disappeared due to oxidation, and two new peaks developed at 1717 cm^−1^ and 1638 cm^−1^. The peak at 1717 cm^−1^ was attributed to the stretching vibration of the C=O of aldehyde groups, and the O-H bending vibration of bound water (in the form of gem-diol) was at 1638 cm^−1^. This was similar to the O-H vibrations of stable chloral hydrate (1625 cm^−1^) [33] and ninhydrin hydrate (1593 cm^−1^) [33] (Figure 7a). This suggested that the vicinal hydroxyl groups in sucrose were oxidized into aldehyde groups and the dried oxidized sucrose contained geminal diol. These results are consistent with the those of LC-MS and HMRS analyses.

### 3.5. H-NMR Analysis of Oxidized Sucrose

**Figure 8:**
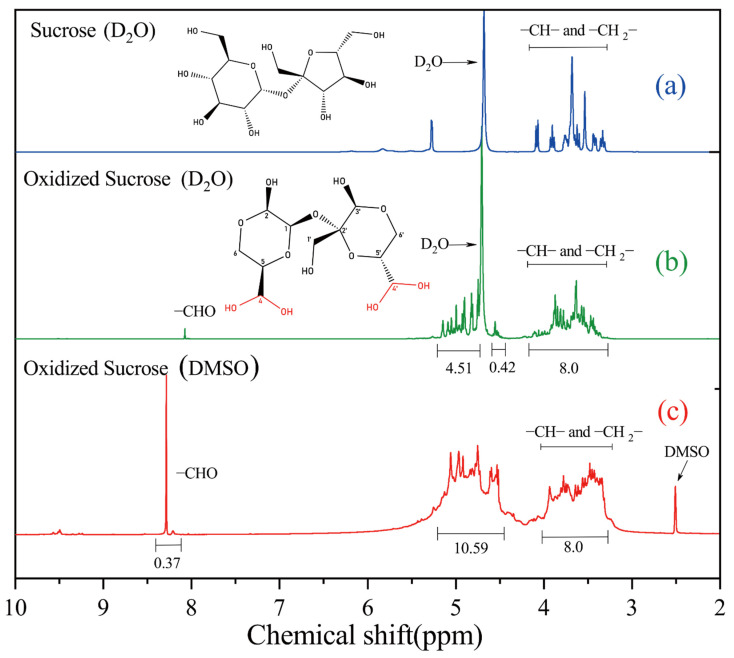
^1^H-NMR spectra of sucrose (D_2_O) (**a**), oxidized sucrose (D_2_O) (**b**) and oxidized sucrose (DMSO) (**c**).

Theoretically, oxidized sucrose in the hemiacetal form contains 13 non-exchangeable protons. Figure 8b shows the ^1^H-NMR spectrum of oxidized sucrose in D_2_O and a normalized integral value. The proton signals from aldehyde groups were barely visible in the NMR spectra. The integral at δ 3.26–4.20 ppm was set as the reference, assuming that it included eight protons, where the carbon contained only one C-O bond (C5, C6, C1′, C5′, C6′). Signals at δ 4.47–5.2 ppm (excluding δ 4.60–4.72 ppm) were assigned to protons containing two C-O bonds (C1, C2, C4, C3′, C4′), and the sum of integral values was 4.93. The chemical shift of the aldehyde group changed after combining with water to form a geminal diol structure. Similarly, the chemical shift of hydrogen on carbon changed from δ 9.8 ppm to δ 5.6 ppm when formylpyridine formed a geminal diol [34]. The integration ratio of two characteristic proton peaks was 8:4.93, and there was a small number of free aldehyde groups; so, this value could be considered consistent with the theoretical value (8:5). The hydroxyl resonances were rarely seen when the ^1^H-NMR spectrum was acquired in D_2_O solution, because the H atoms of −OH groups were rapidly exchanged with ^2^H to form -OD. Therefore, the 1H-NMR spectrum (Figure 8c) was measured in DMSO-d6 solvents. The integral at δ 3.33–4.00 ppm was set as the reference, assuming it included eight protons. The integral value at δ 4.44–5.20 ppm was 10.59. Suppose oxidized sucrose had no geminal diol structure: in that case, each oxidized sucrose molecule contained only three hydroxyl groups, but the integral value increased by 5.59. Hence, the integral value of the hydroxyl group assigned to the geminal diol was about 2.59. At δ 8.29 ppm, we observed that there were still a few free aldehyde groups in the solution. In summary, these results suggested that oxidized sucrose existed in the hydrated form.

### 3.6. Calculation and Theoretical Analysis

**Figure 9:**
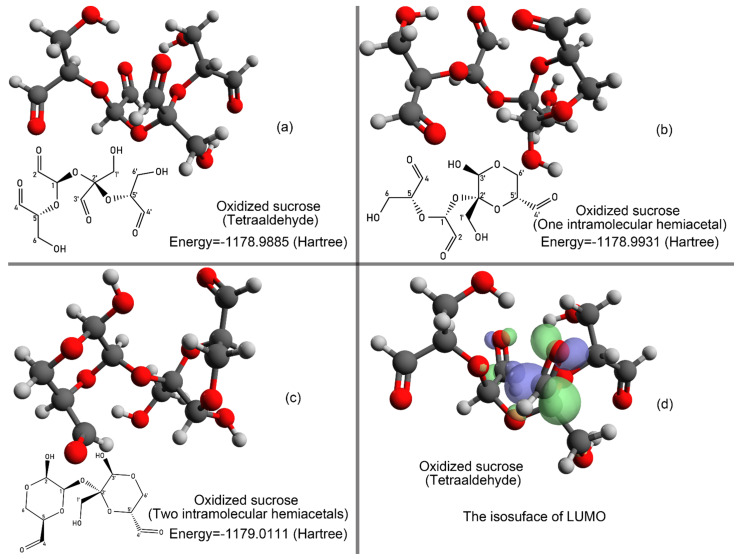
Oxidized sucrose isomers and their energies (**a**–**c**) and visualized LUMO isosurface (**d**) of tetraaldehyde oxidized sucrose.

When polysaccharides are oxidized in aqueous sodium metaperiodate, the aldehyde groups of oxidized sugar residues may form six-membered hemiacetal rings. The lowest energy tetraaldehyde form and the two hemiacetal forms of oxidized sucrose are shown in Figure 9. The free energy of the three oxidized sucrose was −1178.9885 Hartree, 1178.9931 Hartree, and −1179.0111 Hartree. A LUMO isosurface map was obtained by the Avogadro 1.2.0 [35] and POV-Ray 3.7 programs [36]. As shown in Figure 9d, C3′ and C2 were expected to be the most reactive sites to a nucleophilic attack, and C3′ was expected to be more reactive to a nucleophilic attack than C2.

**Figure 10:**
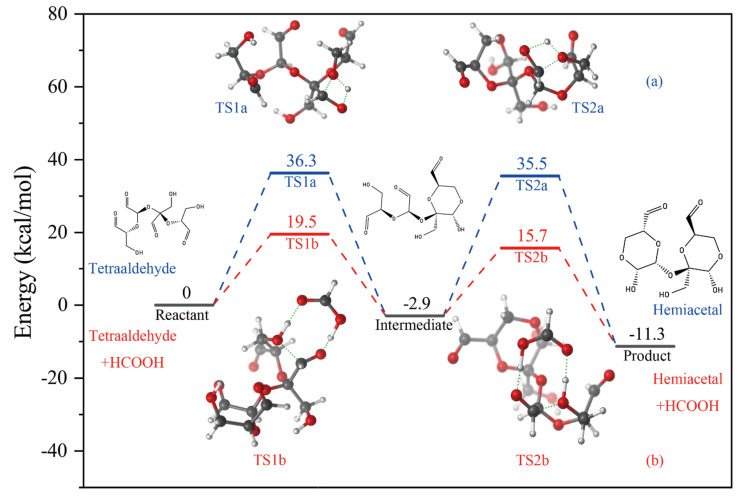
(**a**) Potential energy profile of oxidized sucrose forming a hemiacetal structure. (**b**) Potential energy distribution of oxidized sucrose to form hemiacetal structures assisted by formic acid.

As shown in Figure 10a, the reaction barriers in the process of monomolecular isomerization of tetraaldehyde oxidized sucrose to intramolecular hemiacetal were 36.3 kcal/mol and 38.4 kcal/mol. Generally speaking, the activation free energy was 21 kcal/mol, around the upper limit for reactions that take place easily under room temperature and ambient pressure. Therefore, it was difficult for tetraaldehyde oxidized sucrose to spontaneously isomerize to form hemiacetal at room temperature. As can be seen in Figure 10b, the introduction of formic acid in oxidized sucrose hemiacetalization significantly reduced the activation energy barrier for the unimolecular isomerization step, and the barriers were 19.5 kcal/mol and 18.6 kcal/mol, respectively.

This result suggested that lowering the activation energy barrier was required for the isomerization of tetraaldehyde oxidized sucrose to form an intramolecular six-membered ring hemiacetal. The strong acidity and water removal in the process of extracting oxidized sucrose from the solution reduced the barrier to isomerization, so that the final oxidized sucrose powder had an intramolecular hemiacetal structure. This is consistent with the LCMS characterization results showing that the original solution without impurity removal treatment had four molecular-related ions with different degrees of hydration, and oxidized sucrose did not form a hemiacetal structure at that time. In the HRMS characterization results, oxidized sucrose had formed a stable six-membered ring hemiacetal, which was difficult to reduce to an aldehyde group, and only two free aldehyde groups remained in the molecule and could participate in the hydration reaction. This is consistent with the theoretical analysis results.

### 3.7. Crease-Resistant Application of OS and Its Diffusional Properties in Cotton

**Figure 11:**
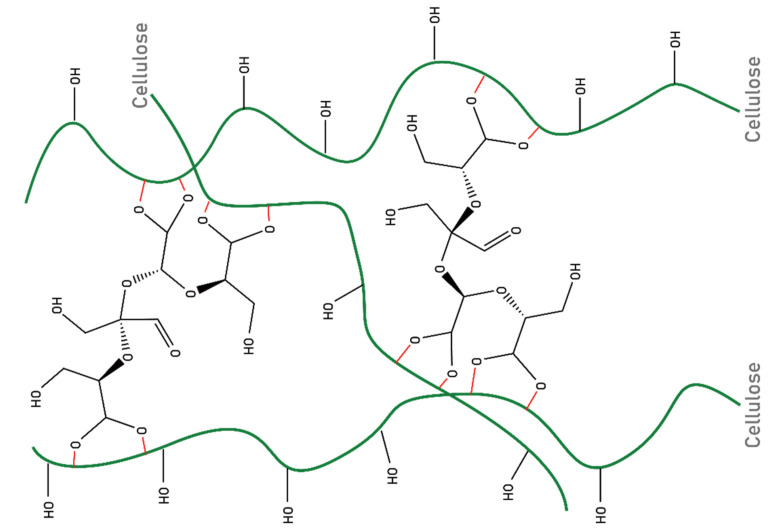
Scheme of cellulose cross-linking with oxidized sucrose.

The treated fabrics were washed five times and stored at a temperature of 20 °C and relative humidity of 65% for 24 h before measurements were performed. The fabric crease recovery angle (CRA) was measured according to Chinese GB/T 3819-1997. The CRA of the control sample was 132°, while the CRA of the treated samples increased to 190°under catalysis with magnesium chloride, and to 260° under catalysis with aluminum chloride. After washing 10 times and 20 times, the CRA of the treated fabrics decreased to 249° and 246°, respectively. The cotton fabrics’ crease resistance was improved significantly. The cross-linking scheme of sucrose and fiber was shown in Figure 11.

During the experiment, it was found that the fabric had to be pretreated by soaking in an oxidized sucrose solution for some time, and then the finished fabric could improve its crease resistance. The improved finishing process achieved similar effects as those obtained with carboxylated polyaldehyde sucrose [37]. Therefore, it was inferred that the diffusion performance of oxidized sucrose was poor, and performing the dipping and rolling process for two times was not enough to make oxidized sucrose diffuse into the cotton fibers. Oxidized sucrose appeared to take a longer time in solution to diffuse into the cotton fibers. Therefore, the rolling-film method was used to analyze the diffusional properties of oxidized sucrose. Cellulose films with aldehydes fully colored the Schiff’s reagent, and the absorbance of the red-violet solution was measured.

**Figure 12:**
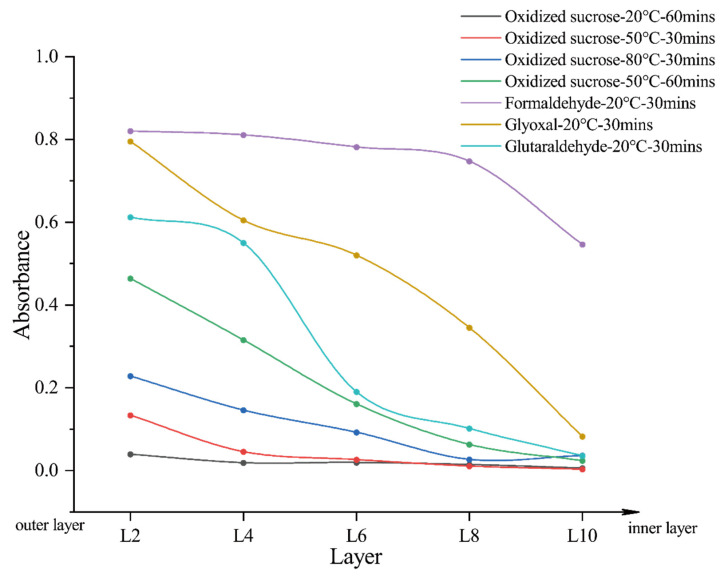
Absorbance of Schiff’s reagent solution stained with cellulose films from outer layer to inner layer (2nd, 4th, 6th, 8th, and 10th layers).

Figure 12 suggests that the aldehyde group content of formaldehyde, glyoxal, and glutaraldehyde in the cellulose roll film was much higher than that in oxidized sucrose at 20 °C. After the solution was heated, the aldehyde group content of oxidized sucrose in the cellulose film increased. However, it was still lower than that of formaldehyde, glyoxal, and glutaraldehyde at 20 °C. Heating was required to improve the diffusional properties of oxidized sucrose. This may be due to the fact that tetraaldehyde oxidized sucrose hydrate contains a large number of hydroxyl groups, which can easily form hydrogen bonds with the hydroxyl groups of cellulose. Therefore, before drying and curing, the cotton fabric should be soaked in a hot oxidized sucrose solution for a while, so that oxidized sucrose can fully diffuse into the fibers. This would noticeably improve the CRA of the cotton fabric.

## 4. Conclusions

In this paper, oxidized sucrose was prepared with sucrose and sodium periodate. The analyses showed that the vicinal diol structure in sucrose was oxidized by sodium periodate to form aldehyde groups. The main oxidative product was OS with tetraaldehyde groups. The aldehyde groups of oxidized sucrose appeared prone to combine with water to form a geminal diol. Oxidized sucrose with tetraaldehyde group could be isomerized to form a double six-membered hemiacetal ring when separated from water under strongly acidic conditions. As a crosslinking agent, oxidized sucrose can effectively improve the crease resistance of cotton fabrics. However, it showed poor diffusional properties. During finishing, the cotton fabric should be immersed in an OS solution for a while to improve the cross-linking.

## Data Availability

The data presented in this study are available on request from the corresponding author.
